# FG-LCNet: A two-stage foreground-guided network for whole-tree litchi counting

**DOI:** 10.1016/j.plaphe.2026.100268

**Published:** 2026-09-07

**Authors:** Yitao Zhuang, Mingchao Yang, Zhikeng Ke, Riyao Chen, Yuefang Gao, Xianghe Wang, Fuchu Hu

**Affiliations:** a Institute of Tropical Fruit Trees, Hainan Academy of Agricultural Sciences, Key Laboratory of Genetic Resources and Utilization of Tropical Fruits and Vegetables (Co-Construction by Ministry and Province), Key Laboratory of Tropical Fruit Tree Biology of Hainan Province, Investigation Station of Tropical Fruit Trees of Ministry of Agriculture, Haikou, 571100, China; b Department of Health Technology and Informatics, The Hong Kong Polytechnic University, Kowloon, Hong Kong Special Administrative Region of China; c College of Mathematics and Informatics, South China Agricultural University, Guangzhou, 510642, China; d School of Automation Science and Engineering, South China University of Technology, Guangzhou, 510641, China

**Keywords:** Litchi counting, Plant phenotyping, Fruit cluster detection, Density map regression, Orchard environments

## Abstract

Accurate litchi counting from whole-tree images is essential for yield estimation, orchard management, and plant phenotyping, but remains challenging in real orchards because fruits occur in dense, heavily occluded clusters and vary markedly in scale, illumination, and appearance across ripening stages, particularly when green fruits resemble surrounding foliage. Existing methods have shown promise, but their robustness in complex orchard environments remains limited. To address these challenges, we propose FG-LCNet, a two-stage foreground-guided litchi counting framework. In the first stage, an enhanced fruit-cluster detector improves the localization of small and ambiguous clusters under complex canopy backgrounds. In the second stage, the detected foreground regions are fed into a density-regression network with hybrid attention, while a consistency-based training strategy is introduced to improve robustness to appearance and illumination variations. To support this study, a large-scale litchi counting dataset was established, consisting of 1126 whole-tree images collected from five orchards and spanning three ripening stages, with approximately 120,000 fruit-level dot annotations and more than 20,000 cluster-level bounding boxes. FG-LCNet achieved the best overall counting performance, with an MAE of 7.44 and an RMSE of 11.01. It showed clear advantages in high-density fruit-cluster scenarios and cross-orchard validation, while maintaining competitive results across orchard-region and maturity-stage subsets. The framework further retained inference efficiency suitable for practical deployment. These results indicate that FG-LCNet provides an effective solution for robust litchi counting and offers potential for other clustered fruit-counting tasks.

## 1 Introduction

Fruit number is a core phenotypic variable that links canopy management, yield estimation, and supply-chain organization [1,2]. China is the world's largest litchi producer, with approximately 524,000 ha under cultivation and contributing about 70% of global output [3,4]. Yet, practical litchi yield estimation still relies primarily on either whole-tree harvesting and weighing, a procedure that is labor-intensive, costly, and unsafe for tall trees, or experience-based visual assessment, which is inherently subjective and often unreliable in dense and heterogeneous canopies [5,6]. These conventional approaches fall short of the high-precision, objective, and consistent fruit counts required by precision agriculture and emerging digital orchard systems [7,8].

Accurate litchi counting in real orchards remains particularly difficult because fruits typically occur in dense, panicle-like clusters and are frequently occluded by foliage [9,10]. In addition to compact and overlapping racemes and small target size at typical working distances, field imagery exhibits strong variability in illumination, motion blur, and viewpoint-induced occlusion. Importantly, during early phenophases, green-stage fruits can be visually confusable with surrounding leaves, further aggravating missed detections and false positives. Together, these factors reduce detection recall, increase ambiguity for instance segmentation and density regression, and weaken the reliability of point-based localization, thereby limiting generalization across orchards, vigor strata, and phenophases.

Early recognition at the green stage is particularly valuable, as early crop-load maps can support proactive thinning and bagging, reduce uncertainty in pre-harvest resource planning (labor allocation, cold-chain capacity, and transportation), and provide early-season yield estimates for market planning and contracting [11,12]. Accordingly, automated methods should deliver stable counts under heavy occlusion and scale variation and remain robust to the appearance of green-stage fruit [13]. This requirement is further reinforced by cultivar-dependent differences in maturation and harvest timing, which motivate maturity-aware perception [14,15].

To replace labor-intensive manual counting in orchards, computer vision and deep learning have been widely explored for automated crop counting [[16], [17], [18], [19], [20]]. Existing litchi-related studies mainly rely on localization-based strategies, including instance segmentation and object detection, while regression-based counting has also been explored for dense local organs. Deka and Chakraborty [21] proposed a UAV-based litchi instance-segmentation pipeline by improving Mask R-CNN with channel attention to enhance robustness under occlusion. Such methods provide fine-grained delineation, but their performance may degrade under severe overlap and foliage interference. Detection-based methods have therefore been more widely adopted for larger scenes. Several studies have developed improved YOLO-based detectors for large-scale or UAV-based litchi detection to enhance recall and robustness under dense fruit distribution, small object size, and mutual occlusion [9,22,23]. Although these methods demonstrate promising localization accuracy and computational efficiency, their reliance on detection may limit counting reliability in cluttered whole-tree scenes, where small or visually ambiguous fruits are prone to be missed. In addition, a panicle-level flower counting framework combining panicle segmentation and density regression has been reported, highlighting the potential of regression-based approaches for highly crowded local organs [24]. However, extending such local-region strategies to whole-tree litchi counting remains challenging because orchard scenes involve stronger background interference, larger scale variation, and maturity-dependent appearance changes. Consequently, robust whole-tree litchi counting under dense clustering, severe occlusion, and phenophase-related appearance variation remains insufficiently explored.

Moreover, existing litchi studies are often evaluated under dataset- or setup-specific protocols and remain fragmented across targets and phenophases, making robustness and reproducible comparison difficult. As a result, robust whole-tree litchi counting that remains reliable under dense clustering, heavy occlusion, and phenophase-related appearance variation is still under-addressed, especially for challenging green-stage scenes. Meanwhile, litchi-specific datasets, standardized evaluation protocols, and method designs tailored to the “clustered fruit + stage shift” setting are comparatively limited, which hinders fair comparison and reproducible progress.

To bridge this gap, we propose FG-LCNet, a foreground-guided two-stage litchi counting framework. In Stage 1, an improved YOLOv11 detector with P2 and Global-to-Local Spatial Aggregation (GLSA) localizes fruit-cluster foreground regions under complex canopy backgrounds. In Stage 2, a hybrid-attention density-regression network performs density-based counting on the background-suppressed images, with a consistency disturbance strategy to improve robustness under field variability. We also construct a large-scale whole-tree litchi counting dataset with cluster-level bounding boxes and fruit-level dot annotations across five orchards and three ripening stages.

Overall, the main contributions of this work can be summarized as follows:

• A foreground-guided two-stage framework, FG-LCNet, is proposed to decouple litchi-cluster localization from within-cluster counting, improving robustness under dense distribution and severe occlusion in real orchards.
• For cluster localization, YOLOv11 is enhanced with a high-resolution P2 head and a lightweight GLSA module, improving small-cluster detection and suppressing background interference under complex canopy conditions.
• For within-cluster counting, a hybrid-attention density-regression network is developed with a consistency disturbance strategy, improving robustness across orchards and ripening stages under field variability.
• A large-scale litchi counting dataset is constructed with both cluster-level bounding boxes and fruit-level dot annotations across five orchards and three ripening stages, providing a benchmark for systematic evaluation and future research.

## 2 Materials and methods

### 2.1 Dataset collection and construction

**Fig. 1:**
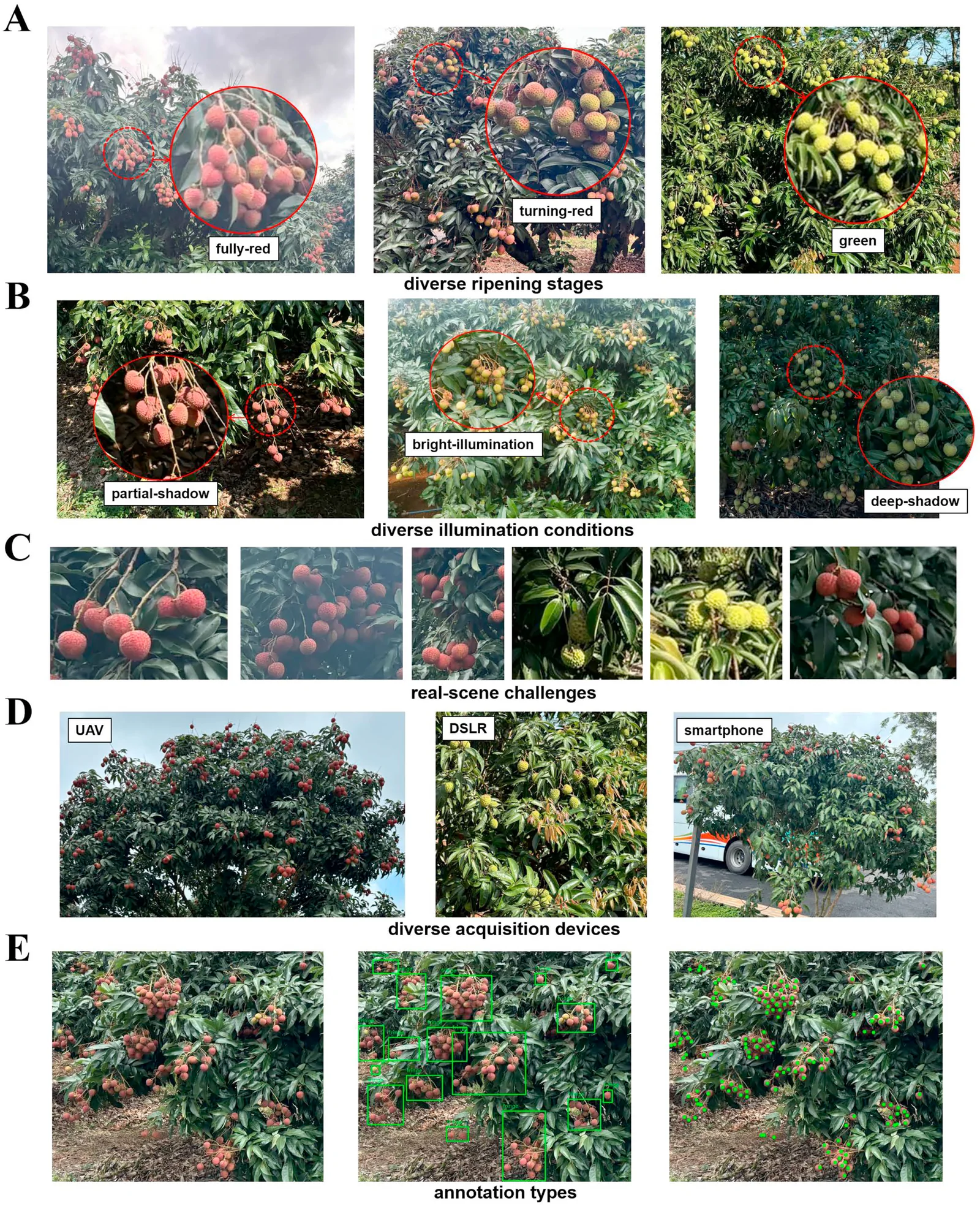
**Overview of the litchi dataset collected under realistic orchard conditions.** (A) Litchi branches at diverse ripening stages, (B) diverse illumination conditions, (C) real-scene challenges such as occlusion and dense fruit distribution, (D) diverse acquisition devices with different fields of view and perspectives, and (E) annotation types in the litchi-counting dataset.

To promote the development of litchi-counting algorithms under realistic orchard conditions, we conducted large-scale image acquisition from February 2023 to August 2024 across five representative orchards located in Haikou, Chengmai, Qionghai, Zhanjiang, and Maoming (denoted as Sites a-e). The dataset encompasses nearly 50 litchi cultivars, including ‘Feizixiao’, ‘Baitangying’, ‘Ziniangxi’, ‘Jinganghongnuo’, and ‘Xiantaoli’, which exhibit substantial variations in fruit morphology and size. To comprehensively capture phenotypic differences, images were collected at three distinct ripening stages—green, turning-red, and fully-red (Fig. 1A).

The data collection was designed to reflect diverse natural illumination and weather conditions, including sunny and overcast days, and various lighting scenarios such as partial shadow, bright illumination, and deep shadow inside the canopy (Fig. 1B). To align with practical yield-estimation requirements, all images depict entire litchi trees rather than individual branches or close-up clusters, thereby introducing significant challenges for automated counting. Here, the basic counting unit is a single whole-tree image, and the ground-truth count refers to the visible litchi fruits annotated from that viewpoint, rather than the complete 3D fruit load of the tree. The fruits are often densely distributed, with severe occlusions and overlaps among clusters, and are frequently partially hidden by leaves and branches (Fig. 1C). Moreover, images were captured using multiple types of devices, including unmanned aerial vehicles (UAVs), digital single-lens reflex (DSLR) cameras, and smartphones (Fig. 1D). UAV images mainly provide upper-canopy views from relatively high viewpoints, DSLR images preserve clearer texture and local fruit details, and smartphone images are commonly acquired from ground-level viewpoints to record whole-tree scenes. These cross-device differences introduce variations in field of view, perspective, and fruit-cluster scale, with image resolutions ranging from 512 × 515 to 2048 × 4846 pixels. This heterogeneity, while increasing the difficulty of detection and counting, more accurately represents real-world orchard conditions and provides a diverse basis for evaluating robustness and practical applicability of litchi-counting algorithms.

For data annotation, we provide both cluster-level bounding boxes and fruit-level dot annotations (Fig. 1E), designed respectively for object detection and density-based counting tasks. The bounding boxes are annotated at the litchi-cluster level, while each dot corresponds to an individual fruit. Each cluster-level bounding box is labeled by the dominant maturity stage of the visible fruits within the cluster. For clusters containing fruits at mixed maturity stages, the label is determined by the predominant stage after annotator consensus. All annotations were manually performed and cross-validated by three independent annotators to ensure labeling accuracy and consistency.

The statistical details of the dataset are summarized in Table 1. As shown, there are significant differences in both the number of fruits and the cluster sizes across regions, and the proportions of different maturity stages also vary noticeably. For instance, Region A is dominated by fully-red fruits, whereas Region C contains a larger proportion of green fruits. Such regional variations and maturity-level imbalance make the dataset not only large in scale but also highly diverse and challenging. Overall, the dataset consists of 1126 images, with approximately 120,000 fruit-level dot annotations and over 20,000 cluster-level bounding boxes. It covers real-world litchi orchard scenes under diverse growth stages, illumination conditions, and environmental settings, providing a large-scale and complex benchmark resource for yield estimation and plant phenotyping research.

**Table 1:** Summary of the litchi dataset.

Region	Image Count	Dot Annotations	Bounding Boxes	Green clusters	Fully-Red clusters	Turning-Red clusters
a	369	47,937	4869	50	4065	754
b	249	20,337	2873	146	1316	1411
c	254	16,801	5528	3559	7	1962
d	109	10,109	2711	36	226	2449
e	145	25,128	5154	279	3540	1335

To evaluate the proposed method, the main dataset was randomly divided into training, validation, and internal testing subsets in a 6:2:2 ratio. This internal image-level split contains samples from all five orchard centers and was used as a mixed-condition evaluation, with comparable overall data distributions across the three subsets. Detailed statistics of the subsets, including the number of images, dot annotations, and bounding boxes, are summarized in Table S1. The experiments reported in Sections 3.1, 3.2, and 3.3.2–3.3.4 were conducted using this internal split. To further assess generalization beyond this mixed split, we designed a cross-center test under unseen orchard conditions: samples from Center e were excluded from the training and validation set, and the testing set was restricted to Center e. The results of this generalization experiment are presented in Section 3.3.5.

In general, the data are relatively balanced across subsets, with each subset covering different regions, maturity stages, and lighting conditions. However, it is worth noting that the number of green fruits is underrepresented, leading to a degree of class imbalance that poses additional challenges for ensuring model robustness across fruit maturity levels.

### 2.2 The proposed FG-LCNet framework

**Fig. 2:**
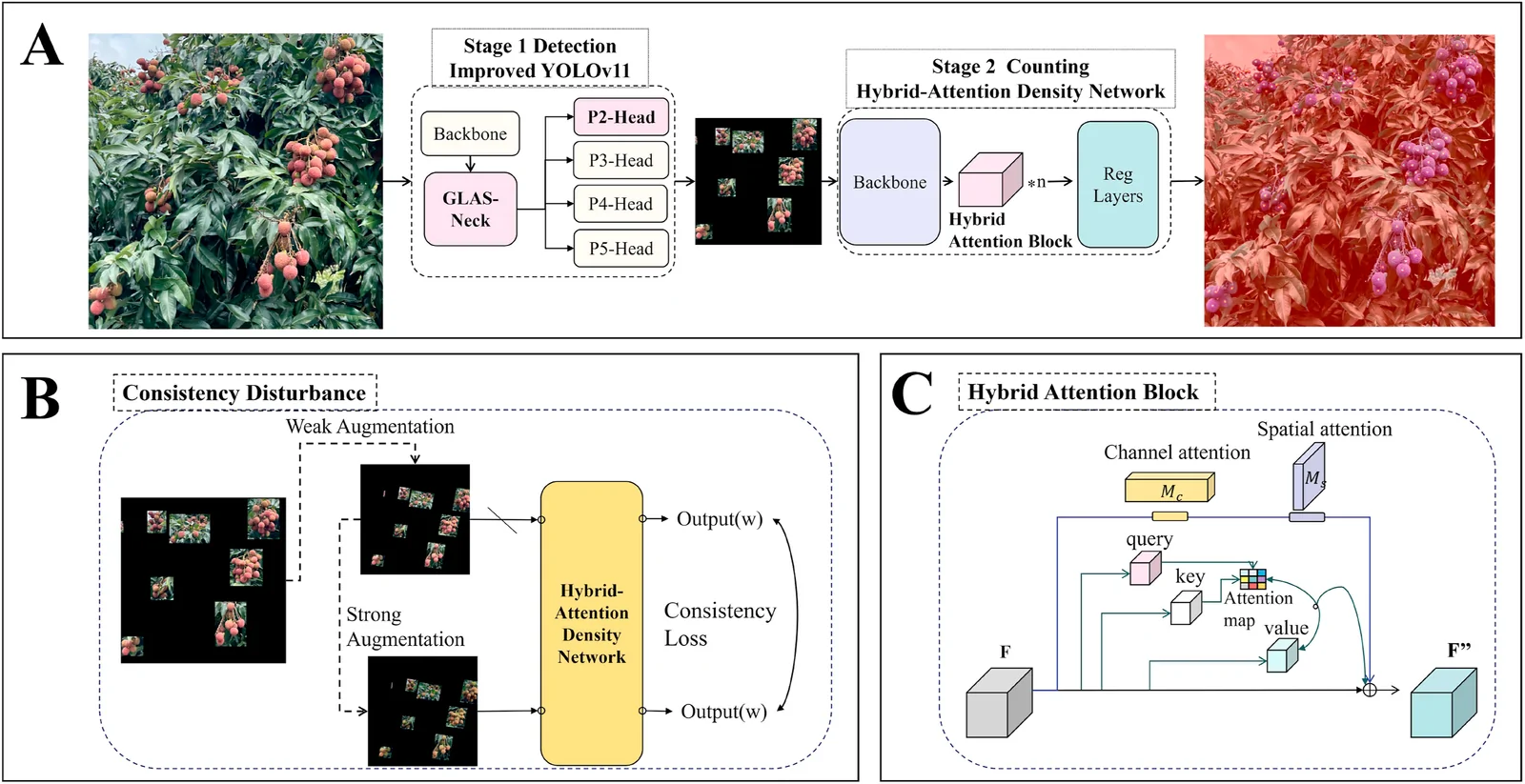
**Overview of the proposed FG-LCNet framework for litchi counting.** (A) The two-stage pipeline of FG-LCNet. (B) The consistency disturbance strategy. (C) The structure of the Hybrid Attention Block.

The overall pipeline of the proposed FG-LCNet is illustrated in Fig. 2. FG-LCNet adopts a foreground-guided two-stage design to address litchi counting in complex orchard scenes. Direct count regression from whole-tree images is easily disturbed by leaves, branches, and illumination noise, whereas fruit-cluster detection alone cannot fully handle dense occlusion and intra-cluster counting ambiguity. Therefore, FG-LCNet first extracts fruit-cluster foreground priors from the whole-tree image and then performs density-based counting on the background-suppressed image. This design reduces irrelevant canopy interference while preserving the spatial layout of fruit clusters for fine-grained counting.

In the first stage, we develop an enhanced object-detection network to localize fruit clusters from tree-level images. The detector incorporates a P2 feature layer to preserve fine spatial cues for small clusters and a GLSA module to enhance global-local contextual discrimination. Together, P2 improves fine-scale sensitivity while GLSA suppresses noisy background responses, leading to more reliable foreground localization. The detector outputs bounding boxes and confidence scores as foreground priors for counting.

In the second stage, the detected fruit-cluster boxes are first aggregated to define foreground regions, and pixels outside these regions are masked out. The resulting background-masked images are then fed into the density-regression network to estimate fruit counts. This counting network is equipped with the proposed hybrid attention module (Fig. 2C), which integrates self-attention with convolutional attention. The module is designed to capture long-range dependencies while preserving local receptive fields, thereby improving the representation of clustered fruit regions.

Furthermore, as shown in Fig. 2B, we introduce a consistency-based supervision strategy: during training, strong data augmentations are applied to the input samples, and the network is required to maintain consistent predictions before and after augmentation. Even in the fully supervised setting, this consistency regularization is intended to improve robustness to appearance perturbations, illumination changes, and local shape variation encountered in orchard scenes.

#### 2.2.1 Stage-1 improved YOLOv11

We employ an improved YOLOv11 [25] as the first-stage detector in our framework. YOLOv11 inherits the efficiency and real-time capability of the YOLO family while introducing several architectural refinements that substantially enhance detection performance. The model follows the standard three-part design, consisting of a Backbone for hierarchical feature extraction, a Neck for multi-scale feature aggregation, and a Head responsible for object localization and classification. YOLOv11 incorporates key modules such as C3k2, SPPF, and C2PSA, which strengthen its representation capacity across diverse spatial structures and object sizes.

**Fig. 3:**
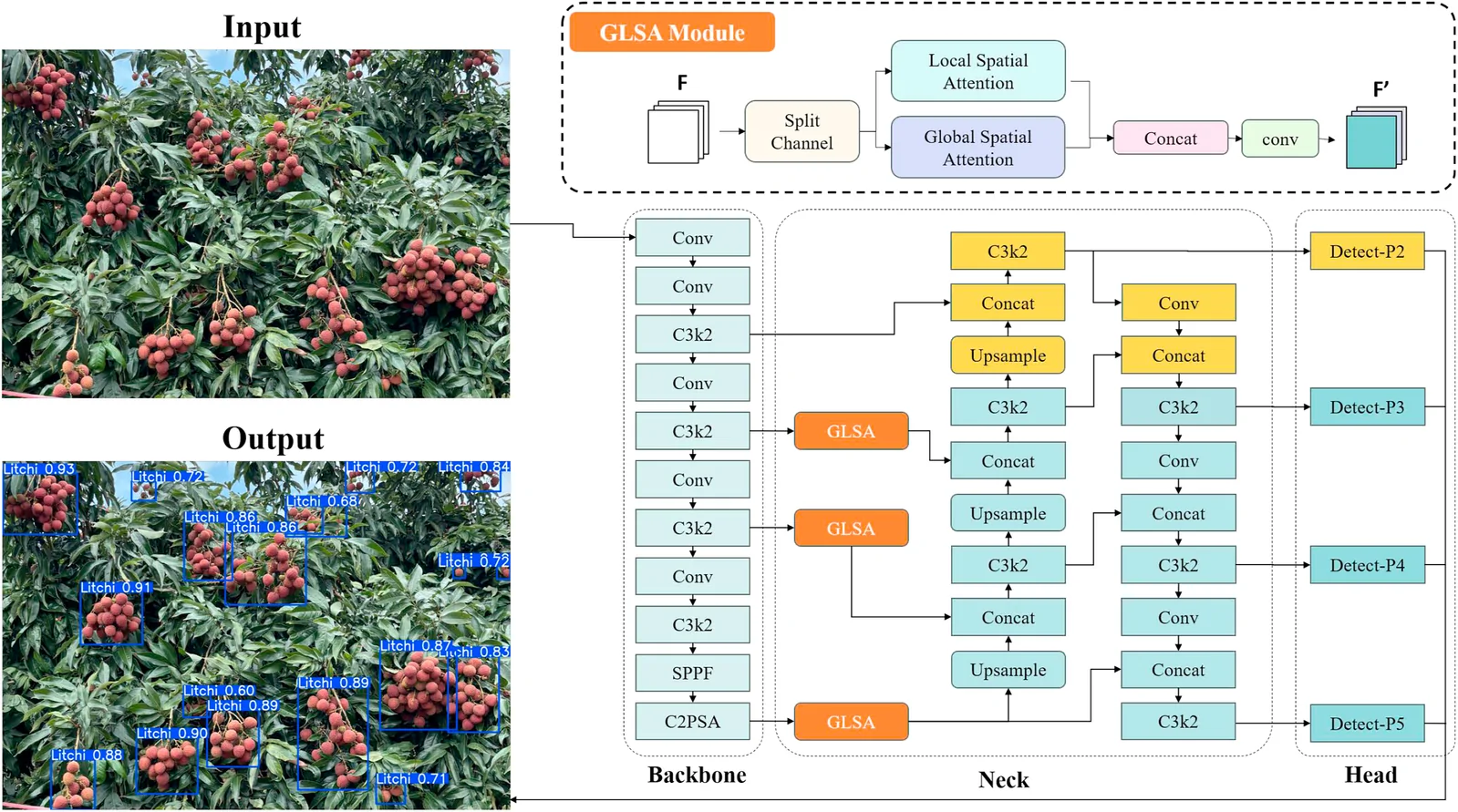
Architecture of the improved YOLOv11 model.

Building upon this foundation, we introduce task-specific adaptations to tailor YOLOv11 for litchi cluster detection (Fig. 3). An additional P2 detection layer and a Global-to-Local Spatial Aggregation (GLSA) module [26] are introduced into the Head and Neck, respectively, to jointly enhance fine-scale cluster sensitivity and context-guided background suppression.

To better accommodate the large number of small and densely packed litchi clusters in real orchard scenes, we extend the original YOLOv11 detection head with an additional high-resolution P2 layer (Fig. 3, yellow block). While the standard YOLOv11 relies on three feature maps (P3/8, P4/16, and P5/32), the newly added P2/4 layer preserves finer spatial details by operating at a higher resolution. This modification helps alleviate the loss of fine spatial detail caused by deep downsampling and is intended to improve sensitivity to small fruit clusters. The resulting four-level feature pyramid (P2/4, P3/8, P4/16, P5/32) enables efficient fusion of deep semantic cues and shallow structural information through cross-scale connections. However, the use of high-resolution shallow features also increases the risk of retaining background responses from leaves, branches, and illumination variations.

To compensate for the noise sensitivity introduced by the high-resolution P2 layer, a GLSA module is incorporated into the Neck to further enhance feature representation (Fig. 3, orange block). The GLSA module adopts a dual-path design that jointly captures global context and local fine-grained details. The global branch employs a ContextBlock with weighted pooling operations to model long-range dependencies, whereas the local branch utilizes a depthwise-separable-convolution residual branch to extract structural details. A spatial attention unit is applied to highlight salient regions, after which the two branches are concatenated and fused via a 1 × 1 convolution. Despite its lightweight design, GLSA provides a mechanism for combining global context and local structural cues in litchi cluster detection. This contextual filtering complements the P2 layer by suppressing distracting responses from leaves, branches, and background textures that may be amplified by high-resolution shallow features.

To mitigate counting errors caused by overlapping detections in Stage 1, we introduce a bounding-box merging strategy. Specifically, all predicted boxes are iteratively examined, and any pair of boxes with an intersection-over-union (IoU) greater than 0 is considered spatially overlapping. These overlapping boxes are then replaced by their minimum enclosing rectangle. The process repeats until no IoU-positive box pairs remain. An illustration of the merging process and its effect in real orchard images is shown in Fig. S1.

#### 2.2.2 Stage-2 Hybrid-Attention Density Network

In the second stage, the detected fruit-cluster boxes are first aggregated to define foreground regions. Background pixels outside these regions are then masked out, and the resulting background-masked images are fed into the proposed Hybrid-Attention Density Network to estimate fruit counts. Compared with using the original whole-tree images or independently cropped patches, this strategy reduces irrelevant background interference while preserving the relative spatial layout and scale relationships among fruit clusters.

**Fig. 4:**
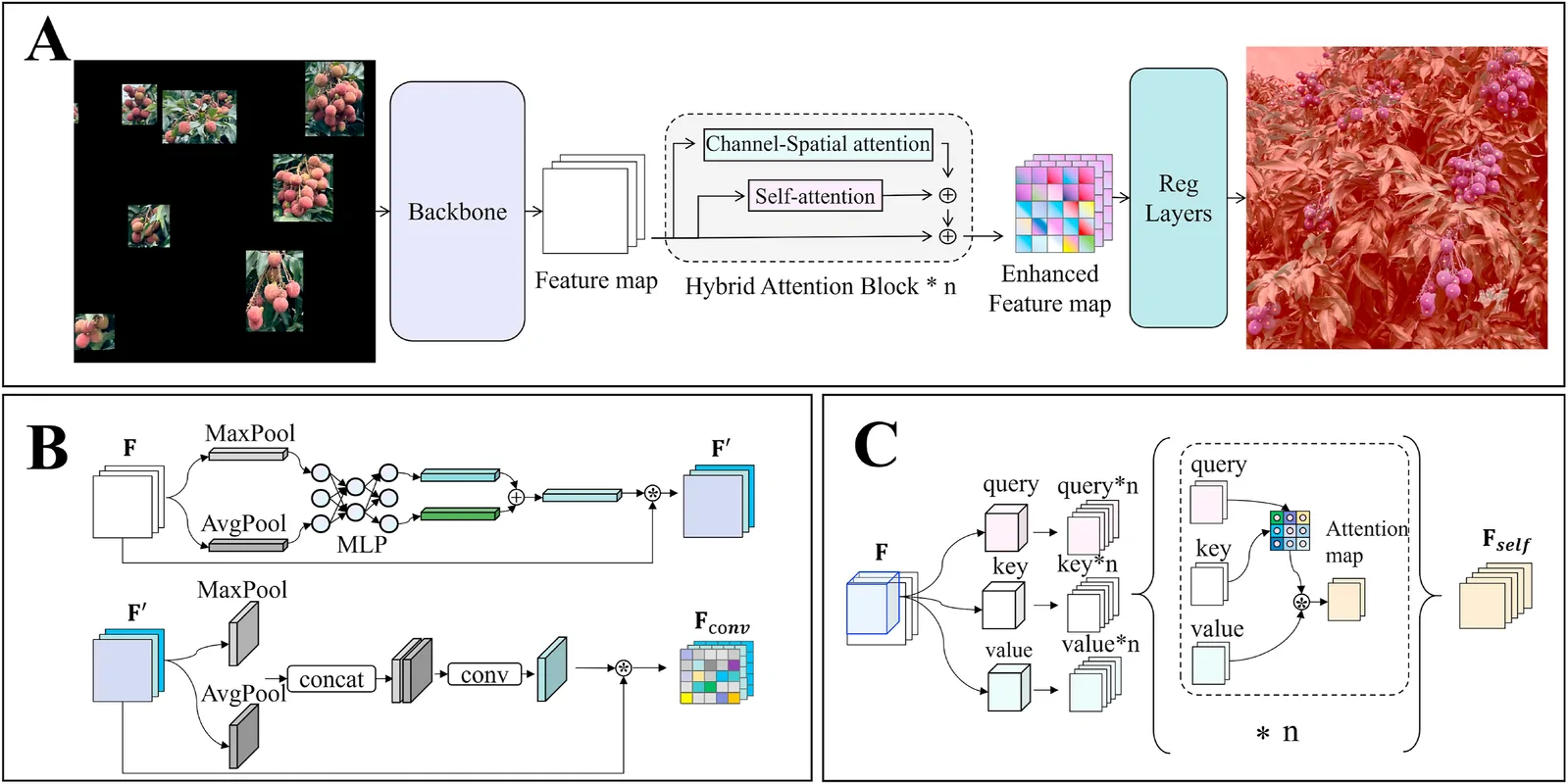
**Overview of the proposed Hybrid-Attention Density Network.** (A) The overall pipeline of the proposed network. (B) The architecture of the spatial–channel attention block. (C) The architecture of the self-attention block.

As illustrated in Fig. 4A, the second-stage model consists of three major components: a feature extraction backbone, the hybrid attention block stack, and a density regression head. The hybrid attention blocks combine self-attention and convolutional attention in a complementary manner to enhance global contextual modeling and local feature representation.

The front-end backbone adopts VGG19, which extracts hierarchical semantic features through sequential convolution and pooling operations. Compared with lightweight backbones, VGG19 provides richer texture and boundary information, which is beneficial for distinguishing fine-grained differences among adjacent fruits.

In the intermediate stage, several Hybrid Attention Blocks are stacked. Each block contains two complementary branches: a self-attention branch that captures global contextual dependencies within foreground fruit regions, and a convolutional-attention branch that enhances local spatial–channel cues and fine-grained fruit details. This hybrid design is intended to enhance robustness to variation in illumination, occlusion, and fruit morphology, while the detailed structure of the Hybrid Attention Module is described in the following subsection.

The final regression head consists of three convolutional layers that gradually reduce channel dimensionality and project the refined features into a single-channel density map. Each pixel value in the predicted density map corresponds to the local fruit density, and the integral of the density map yields the estimated fruit count within the foreground regions. The entire network is trained in an end-to-end manner, with the hybrid attention mechanism facilitating the integration of multi-scale and multi-level information for litchi fruit counting.

#### 2.2.3 Hybrid attention module

The Hybrid Attention Module is inserted into the intermediate features of the density-regression network to balance representation capacity and computational efficiency. This placement avoids the high cost of applying attention to input images or density maps, while retaining useful semantic and spatial information for counting. The module captures global contextual dependencies among foreground fruit regions and preserves fine-grained local cues. These cues are important for distinguishing densely adjacent and partially occluded litchis during density regression.

Inspired by recent efforts that combine convolutional representations with attention-based context modeling [27], the module consists of two parallel branches: a self-attention branch for contextual dependency modeling and a convolutional-attention branch for emphasizing locally informative structures. Their outputs are fused with learnable weights and combined with a residual connection, yielding a unified representation that balances contextual perception and local feature enhancement.

The convolutional-attention branch is built on the CBAM mechanism [28], where channel attention and spatial attention are sequentially applied to refine local feature representations (Fig. 4B). Given an input feature map $F\in R^{B\times C\times H\times W}$, the channel attention module first applies global average pooling and max pooling to produce two channel descriptors, **F**_avg_ and $F_{\text{max}}\in R^{B\times C\times 1\times 1}$. These descriptors are passed through a shared two-layer multilayer perceptron (MLP), where the first layer reduces the channel dimension to *C*/*r* and the second restores it to *C*. The outputs of the two branches are summed and activated by a sigmoid function to generate the channel attention weights:

(1) $$\begin{array}{ll} M_{c}\left(F\right) & =\sigma \left(MLP\left(F_{\text{avg}}\right)+MLP\left(F_{\text{max}}\right)\right)\\ & =\sigma \;\left(W_{1}\;\delta \left(W_{0}F_{\text{avg}}\right)+W_{1}\;\delta \left(W_{0}F_{\text{max}}\right)\right), \end{array}$$

where *σ* denotes the sigmoid function, and **W**_0_ and **W**_1_ are shared MLP weights. The refined feature map is obtained by channel-wise multiplication:

(2) $$F^{\prime }=M_{c}\left(F\right)⊗F,$$

where ⊗ denotes element-wise multiplication with broadcasting.

The spatial attention module takes **F**′ as input and performs average pooling and max pooling along the channel dimension to obtain two spatial descriptors, $F_{\text{avg}}^{s}$ and $F_{\text{max}}^{s}\in R^{B\times 1\times H\times W}$. These descriptors are concatenated and passed through a 7 × 7 convolution followed by a sigmoid activation to produce the spatial attention weights:

(3) $$\begin{array}{ll} M_{s}\left(F^{\prime }\right) & =\sigma \;\left(f^{7\times 7}\;\left(\left[AvgPool\left(F^{\prime }\right);\;MaxPool\left(F^{\prime }\right)\right]\right)\right)\\ & =\sigma \;\left(f^{7\times 7}\;\left(\left[F_{\text{avg}}^{s};\;F_{\text{max}}^{s}\right]\right)\right), \end{array}$$

where *f*^7×7^(⋅) denotes a convolution with a 7 × 7 kernel. The output of the convolutional-attention branch is then

(4) $$F_{\text{conv}}=M_{s}\left(F^{\prime }\right)⊗F^{\prime }.$$

The self-attention branch is inspired by the local self-attention design of Ramachandran et al. [29]. In this work, attention computation is restricted to a fixed local window (with window size *k* = 7) to capture contextual dependencies within a neighborhood while controlling computational cost (Fig. 4C). Given the input feature map $F\in R^{B\times C\times H\times W}$, the query (Q), key (K), and value (V) features are first generated through three independent 1 × 1 convolutions:

(5) $$Q=W_{Q}*F,\;K=W_{K}*F,\;V=W_{V}*F.$$

The channel dimension is divided into *h* attention heads, each with dimensionality *d*_*h*_ = *C*/*h*. For the *m*-th head, local attention is computed within the neighborhood $N_{k}\left(i\right)$ centered at position *i*. The attention score between query position *i* and key position *j* is defined as

(6) $$S_{ij}^{\left(m\right)}=\frac{{\left(Q_{i}^{\left(m\right)}\right)}^{⊤}\left(K_{j}^{\left(m\right)}+P_{i}^{\left(m\right)}-P_{j}^{\left(m\right)}\right)}{\sqrt{d_{h}}},$$

where $P_{i}^{\left(m\right)}$ and $P_{j}^{\left(m\right)}$ are learnable positional embeddings, and their difference provides a relative positional cue for local attention.

The normalized local attention weight is obtained by Softmax:

(7) $$A_{ij}^{\left(m\right)}=\frac{\exp\left(S_{ij}^{\left(m\right)}\right)}{\underset{j\in N_{k}\left(i\right)}{\sum }\exp\left(S_{ij}^{\left(m\right)}\right)},$$

and the weighted aggregation of local features for the *m*-th head is

(8) $${head}_{m}\left(i\right)=\underset{j\in N_{k}\left(i\right)}{\sum }A_{ij}^{\left(m\right)}\;V_{j}^{\left(m\right)}.$$

All heads are concatenated and linearly projected to form the self-attention output:

(9) $$F_{\text{self}}=Concat\left({head}_{1},\ldots ,{head}_{h}\right)\;W_{O}.$$

Finally, the outputs of the two branches are fused with learnable weights and combined with the residual connection:

(10) $$F_{\text{HA}}=F+a\;F_{\text{self}}+b\;F_{\text{conv}},$$

where *a* and *b* are learnable fusion weights. In this way, the Hybrid Attention Module provides context-enhanced and locally refined feature representations for subsequent density regression.

#### 2.2.4 Loss function

For the fruit-detection branch of FG-LCNet, we keep its default composite detection loss [25], which combines IoU- and DFL-based bounding-box regression with BCE-based classification/objectness terms.

In contrast, for the density-map regression branch, we design a joint loss that combines a distribution-alignment loss with a consistency loss. This joint objective constrains both the spatial alignment of the predicted density maps and the robustness of the model under varying imaging conditions. The total loss is defined as

(11) $$L_{\text{total}}=\frac{1}{2}e^{-s_{1}}\;L_{\text{sup}}+\frac{1}{2}e^{-s_{2}}\;L_{\text{cons}}+\frac{1}{2}\left(s_{1}+s_{2}\right),$$

where $L_{\text{sup}}$ denotes the supervised loss that measures the discrepancy between the predicted density map and the ground-truth annotations, and $L_{\text{cons}}$ is the consistency loss that enforces stable predictions before and after data augmentation. The trainable scalars *s*_1_ and *s*_2_ are uncertainty parameters [30] that automatically balance the two loss terms during optimization.

To measure the discrepancy between the predicted density map and the ground-truth annotation distribution, we employ the unbalanced optimal transport (UOT) loss proposed by Wan et al. [31] as our supervised loss. The UOT loss directly computes the transportation cost between the predicted continuous density map and the discrete ground-truth annotation points, providing a principled way to quantify their spatial mismatch.

Let the predicted density map be denoted as $A={\left\{\left(a_{i},x_{i}\right)\right\}}_{i=1}^{n}$ and the ground-truth annotation set as $B={\left\{\left(b_{j},y_{j}\right)\right\}}_{j=1}^{m}$. Here, *a*_*i*_ denotes the predicted density value at pixel location **x**_*i*_, and *n* is the total number of pixels. Similarly, *b*_*j*_ denotes the annotated litchi count at the ground-truth location **y**_*j*_, and *m* is the total number of annotation points.

The UOT loss is formulated as

(12) $$L_{\text{T}}^{c}\left(A,B\right)=\min_{P\in R_{+}^{n\times m}}\left\langle C,P\right\rangle -\epsilon H\left(P\right)+\tau D_{1}\left(P1_{m}\|a\right)+\tau D_{2}\left(P^{⊤}1_{n}\|b\right),$$

Here, **C**_*ij*_ denotes the transport cost between pixel location **x**_*i*_ and annotation point **y**_*j*_, and **P** is the transport plan matrix. The term $\min_{P\in R_{+}^{n\times m}}\left\langle C,P\right\rangle$ represents the minimal transportation cost required to move the predicted density mass to the ground-truth annotation points. The entropy regularization term −*ɛH*(**P**) encourages smoother transport plans and speeds up the convergence of the Sinkhorn iterations. The term *τD*_1_(**P1**_*m*_‖**a**) measures the *L*_1_ discrepancy between the density map reconstructed from the ground-truth annotation points and the predicted density map. Similarly, *τD*_2_(**P**^*⊤*^**1**_*n*_‖**b**) measures the *L*_2_ discrepancy between the point distribution reconstructed from the predicted density map and the ground-truth annotation points.

To further enhance robustness, we introduce a consistency loss inspired by recent advances in semi-supervised learning [32,33], and adapt the strong–weak augmentation paradigm to our fully supervised litchi counting task. This design encourages the model to produce invariant predictions under geometric and photometric perturbations, thereby improving generalization to varying illumination, occlusion, and viewpoint conditions.

Given an input image **x**, we first apply a set of relatively mild geometric augmentations (e.g., random horizontal flipping, scaling, translation, and shearing) to obtain a weakly perturbed sample **x**_weak_. Based on this sample, we then apply additional strong non-geometric perturbations such as color jitter, brightness variation, and Gaussian blurring to generate a strongly augmented sample **x**_strong_. The network processes both views and outputs the corresponding density maps ${\hat{D}}_{\text{weak}}=f\left(x_{\text{weak}}\right)$ and ${\hat{D}}_{\text{strong}}=f\left(x_{\text{strong}}\right)$.

To enforce prediction consistency before and after augmentation, we adopt the mean squared error (MSE) as the constraint:

(13) $$L_{\text{cons}}=\frac{1}{N}\overset{N}{\underset{i=1}{\sum }}{\left\|f\left(x_{\text{weak}}^{\left(i\right)}\right)-f\left(x_{\text{strong}}^{\left(i\right)}\right)\right\|}_{2}^{2},$$

where (⋅) denotes the counting network, and *N* is the batch size. This loss encourages stable responses in the prediction space with respect to input perturbations, reducing the model's sensitivity to specific imaging conditions and improving its stability under variations in lighting, viewpoint, and occlusion. Unlike consistency regularization that is commonly used in semi-supervised settings, we incorporate it here into a fully supervised density-regression framework to improve prediction stability under heterogeneous orchard conditions.

#### 2.2.5 Training strategy

Algorithm 1 summarizes the overall two-stage training procedure of FG-LCNet. In Stage 1, a fruit detector *f*_det_ is first trained on the detection subset $I_{\text{det}}$ with bounding-box annotations B. For each mini-batch (*I*, *B*^gt^), the detector produces predictions ${\hat{Y}}_{\text{det}}$ and is optimized using the standard detection loss $L_{\text{det}}$, with parameters *θ*_det_ updated by gradient descent. After convergence, the trained detector is applied to the counting subset $I_{\text{cnt}}$ to obtain predicted fruit boxes *B*_pred_, which are further merged into aggregated boxes *B*_agg_. The resulting images, point annotations, and aggregated boxes are then stored in an intermediate dataset D for foreground-guided density regression.

In Stage 2, the density-map network *f*_den_ is trained on the constructed dataset D. For each mini-batch (*I*_*b*_, *P*_*b*_, *B*_*b*_), the aggregated boxes *B*_*b*_ are used to mask out background pixels in *I*_*b*_, yielding background-masked input images *X*_*b*_. The corresponding density targets *G*_*b*_ are generated from the point annotations *P*_*b*_ within the same foreground regions. The supervised branch directly regresses a density map ${\hat{G}}^{\text{sup}}$ from *X*_*b*_, and the prediction is constrained by the unbalanced optimal transport loss $L_{\text{sup}}$. In parallel, a strong–weak consistency branch is introduced: a weakly perturbed view $X_{b}^{\text{w}}=T_{\text{w}}\left(X_{b}\right)$ is first generated, and a strongly perturbed view $X_{b}^{\text{s}}=T_{\text{s}}\left(X_{b}^{\text{w}}\right)$ is then constructed by applying stronger perturbations on top of the weak view. The weak-view prediction ${\hat{G}}^{\text{w}}$ is treated as a stop-gradient target, while the strong-view prediction ${\hat{G}}^{\text{s}}$ is optimized to match it through a mean squared error consistency loss $L_{\text{cons}}$. Finally, the overall density-regression loss $L_{\text{den}}$ is obtained by combining $L_{\text{sup}}$ and $L_{\text{cons}}$ with an uncertainty-based weighting function $L_{\text{comb}}\left(\cdot \right)$, and the parameters *θ*_den_ are optimized by gradient descent.

Algorithm 1 Training procedure of FG-LCNet

**Figure:**
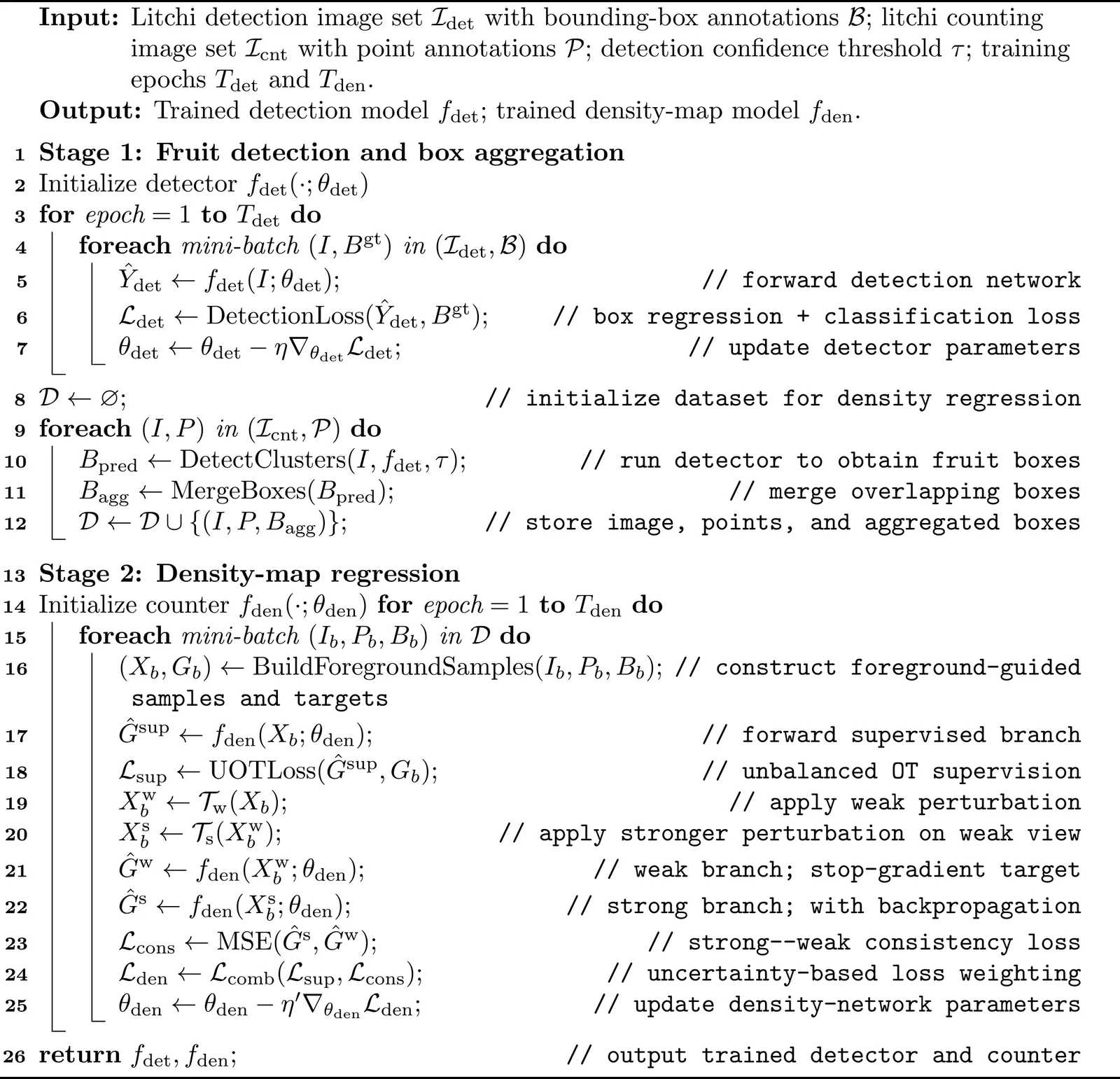

##### 2.2.5.1 Implementation details

The proposed FG-LCNet is implemented in PyTorch. For Stage 1, the improved YOLOv11 detector is trained following the default Ultralytics training settings. During Stage-1 inference, the detection confidence threshold *τ* is fixed at 0.25 to filter low-confidence fruit-cluster proposals before bounding-box merging. For Stage 2, the density-regression network is optimized using Adam with an initial learning rate of 1 × 10^−5^ and a weight decay of 1 × 10^−5^. The background-masked input images are randomly cropped to 256 × 256 to match the network input size and enhance sample diversity. The batch size is set to 16, and the network is trained for 1000 epochs. All experiments are conducted on a single NVIDIA RTX 3090 GPU.

Within the density-regression network, three Hybrid Attention Blocks are inserted into the intermediate layers of the backbone. In each block, the local self-attention window size is set to kernel_att = 7, and the number of attention heads is set to head = 4.

##### 2.2.5.2 Evaluation metrics

To comprehensively evaluate the proposed two-stage framework, we adopt different metrics for the detection and counting stages.

For the Stage-1 fruit-cluster detector, AP_50_ is used to measure the overall cluster-detection performance at an IoU threshold of 0.5. In addition, HitRate_green_ is reported as a supplementary cluster-level metric for green-stage cluster localization, because green fruits are visually similar to surrounding foliage and are more prone to missed detection. Specifically, *N*_g_ denotes the number of annotated green-stage cluster boxes, and *M*_g_ denotes the number of correctly localized green-stage clusters, defined as those matched by at least one predicted cluster box with IoU >0.5. The green-stage cluster hit rate is computed as:

(14) $${HitRate}_{\text{green}}=\frac{M_{\text{g}}}{N_{\text{g}}}.$$

This metric is used only to assess detector sensitivity to green-stage clusters, rather than fruit-level maturity classification or maturity-specific counting accuracy.

For final counting performance, Mean Absolute Error (MAE) and Root Mean Squared Error (RMSE) are used as the primary metrics. Given *N* test images, with *y*_*i*_ and ${\hat{y}}_{i}$ denoting the ground-truth and predicted litchi counts of the *i*-th image, respectively, MAE and RMSE are defined as:

(15) $$MAE=\frac{1}{N}\overset{N}{\underset{i=1}{\sum }}\left|{\hat{y}}_{i}-y_{i}\right|,$$

(16) $$RMSE=\sqrt{\frac{1}{N}\overset{N}{\underset{i=1}{\sum }}{\left({\hat{y}}_{i}-y_{i}\right)}^{2}}.$$

The same counting metrics are used for both overall evaluation and subset-level analyses.

## 3 Results

### 3.1 Fruit detection performance

Among the evaluated detectors, the proposed method achieved the best fruit-cluster detection performance, outperforming several YOLO variants [25,34,35] and the transformer-based DETR-style model RT-DETR [36], with the highest AP_50_ and competitive HitRate_green_ (Table 2(i)). These results indicate accurate localization of fruit clusters while preserving sensitivity to green-stage, less mature fruits. The improvement can be largely attributed to the P2 detection layer and the GLSA-enhanced feature representation, which better capture small-scale targets and mitigate appearance ambiguity in real orchard scenes.

**Table 2:** Performance comparison on the litchi counting benchmark.

(i) Stage-1 cluster detection		
Method	*AP*_50_*↑*	HitRate_green_*↑*
YOLOv8	0.743	57.40%
YOLOv10	0.725	57.35%
YOLOv11	0.760	62.02%
RT-DETR	0.700	**68.43%**
Ours	**0.769**	68.07%

(ii) Whole-tree counting		
Method	MAE *↓*	RMSE *↓*
BL	9.89	21.71
DM-Count	8.76	12.76
GL	9.54	20.62
Gramformer	10.57	22.79
MS2	10.72	19.64
Ours	**7.44**	**11.01**

### 3.2 Overall counting performance

#### 3.2.1 Quantitative comparison

The proposed method achieved the best overall counting performance among all compared approaches, with the lowest MAE and RMSE (Table 2(ii)). Specifically, comparisons were conducted against several representative and widely adopted counting models, including BL [37], DM-Count [38], GL [31], Gramformer [39], and MS2 [40]. The results demonstrate more accurate and stable counting under complex orchard conditions, which is likely related to the foreground-guided two-stage design and the hybrid attention-based density regression network. Furthermore, these results suggest that explicitly decoupling detection and counting, together with global–local feature modeling, is beneficial for fruit counting in complex orchard environments.

#### 3.2.2 Qualitative visualization of the proposed two-stage pipeline

**Fig. 5:**
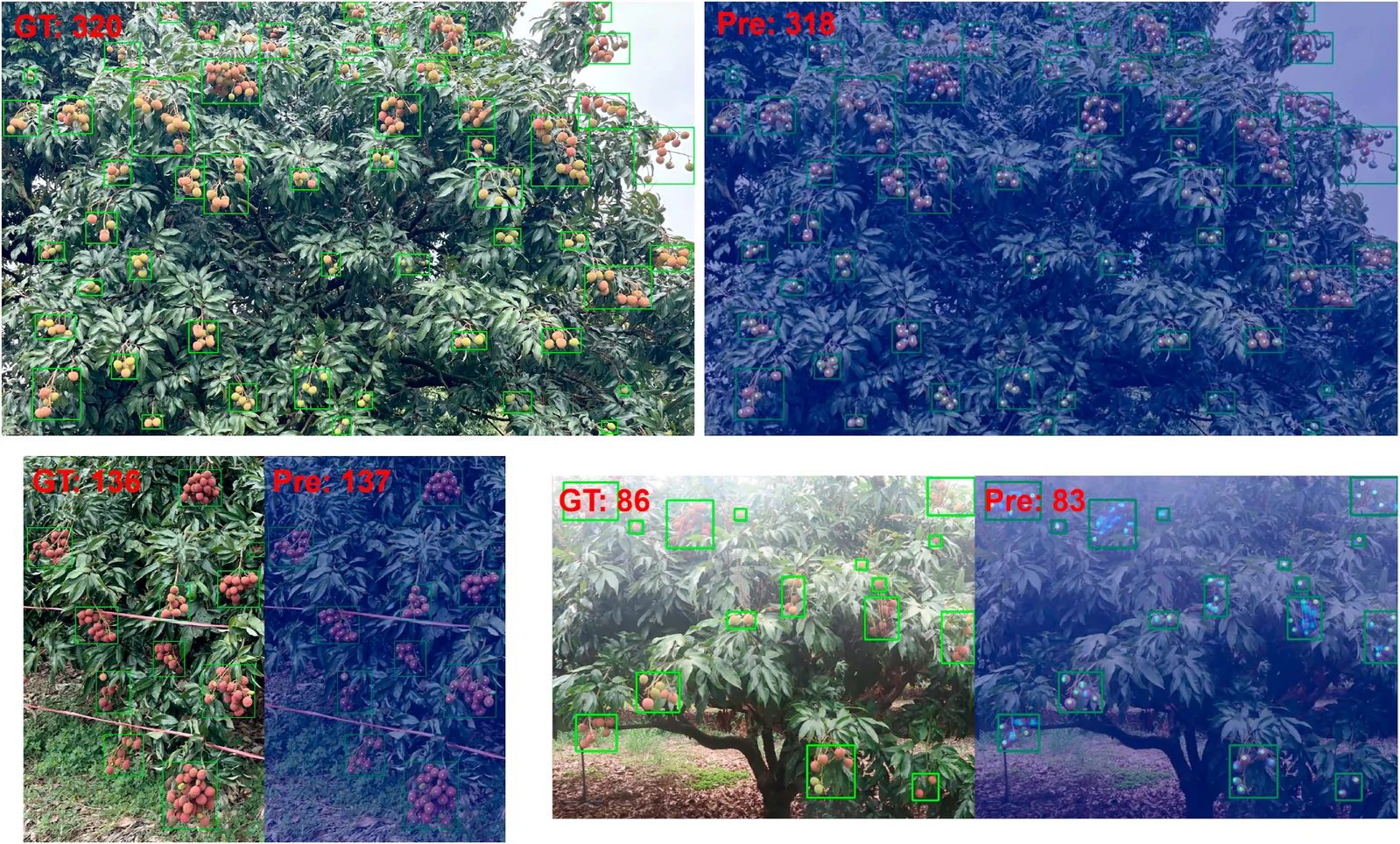
Visualization of fruit cluster detection and counting results of the proposed method.

Representative predictions under diverse orchard conditions are presented in Fig. 5. For each example, the left panel shows the RGB image with Stage-1 detected fruit-cluster bounding boxes, together with the ground-truth count (GT). The right panel overlays the predicted density map within these detected clusters, reporting the final predicted count (Pre) obtained by integrating the density values within the foreground regions. Visual inspection confirms that the proposed method maintains strong consistency between predicted and ground-truth counts. This performance holds even in challenging scenarios characterized by dense raceme-like clusters, severe foliage occlusion, and strong illumination variations, highlighting the practical robustness of the detection–counting decoupling strategy in complex canopy environments.

#### 3.2.3 Qualitative comparison with mainstream density-map counting models

**Fig. 6:**
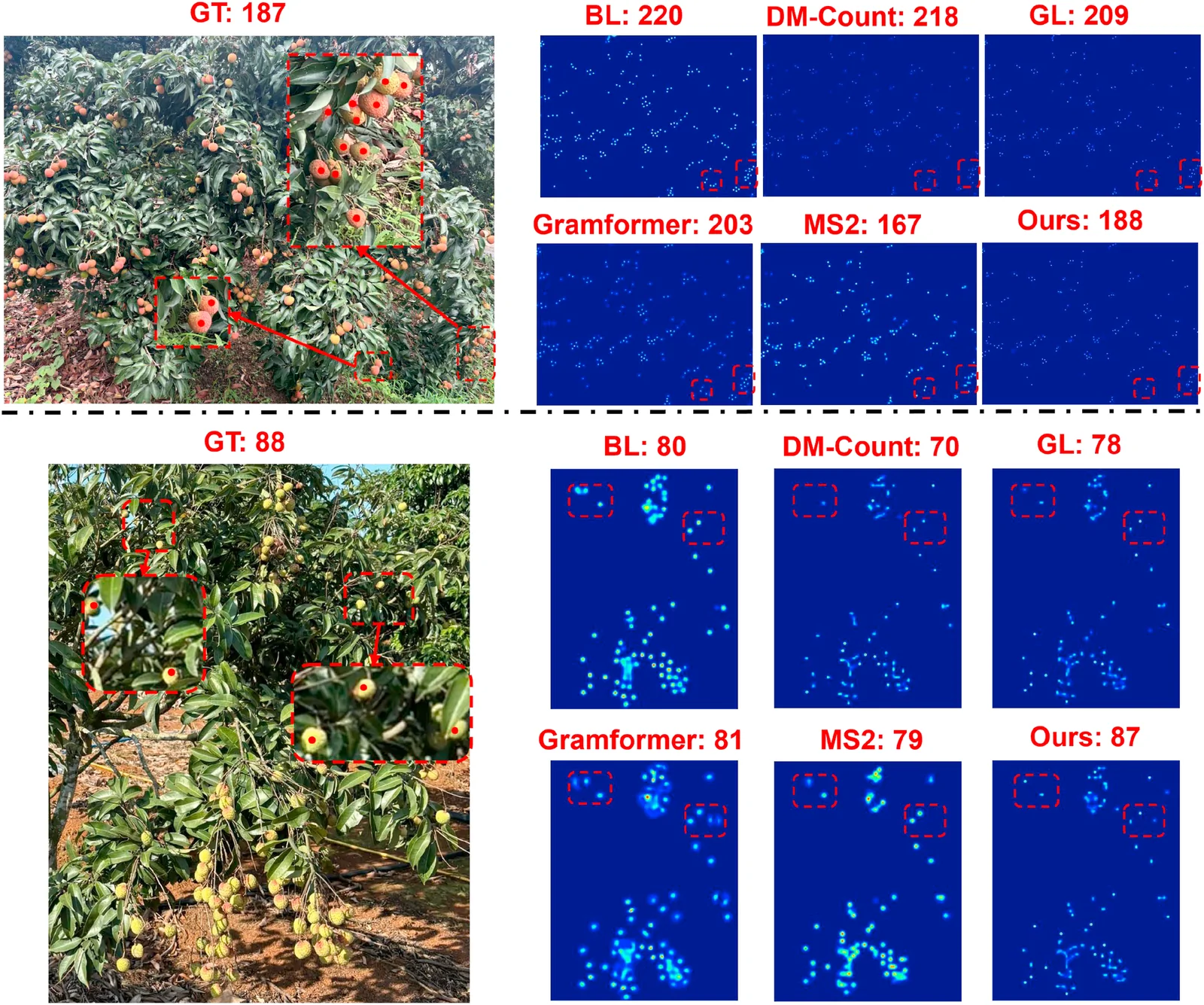
Qualitative comparison of density-map-based fruit counting results.

Qualitative comparisons of density-map predictions on two representative orchard scenes highlight the robustness of the proposed approach (Fig. 6). To facilitate visual inspection, the left column of each example displays the input RGB image annotated with the ground-truth count (GT), together with zoom-in views of selected challenging regions. The right columns visualize the corresponding density maps predicted by different methods, where the red numbers denote the final predicted counts obtained by integrating the density maps, and the dashed boxes indicate representative regions for closer inspection.

Several baseline models tend to produce spurious density responses on cluttered foliage or textured backgrounds, leading to over-counting in dense scenes (Fig. 6, top row). Conversely, these models frequently miss weak or small targets under occlusion or early green-stage appearance (bottom row), resulting in underestimation (Fig. 6, bottom row). In contrast, the proposed method mitigates these issues by generating density maps that are more foreground-concentrated and spatially compact. By minimizing background activations and improving alignment with actual fruit locations, the proposed predictions consistently align more closely with the ground-truth counts across both dense, ripe clusters and small, ambiguous targets. This provides strong visual evidence for the effectiveness of integrating foreground-guided detection with global–local density regression.

### 3.3 Stratified and cross-orchard analysis of counting results

#### 3.3.1 Overview

**Fig. 7:**
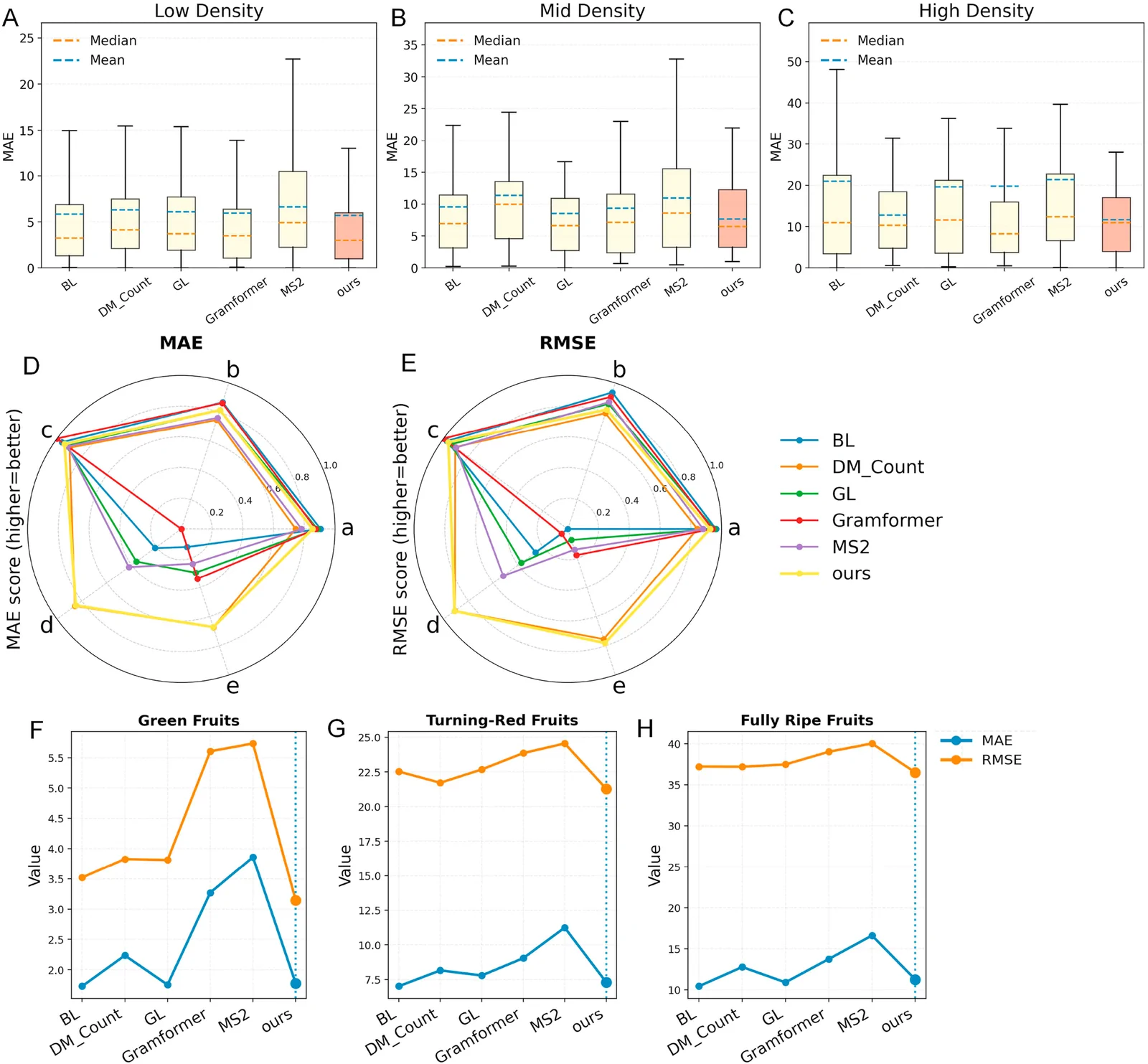
**Density- and region-stratified counting performance.** (A-C) MAE distribution under low (A), medium (B), and high (C) fruit density (box plots; dashed lines indicate mean and median). (D-E) Region-wise normalized MAE (D) and RMSE (E) scores (radar plots; higher values indicate better performance). (F-H) Counting performance across fruit maturity stages: Green (F), Turning-red (G), and Fully-red (H), reported in terms of MAE and RMSE for different counting methods on the corresponding subsets.

To better understand model performance under diverse orchard conditions, a stratified evaluation was conducted on the test set from three complementary perspectives: (1) region-centered analysis, (2) density-centered analysis, and (3) maturity-centered analysis. Specifically, the region split follows different orchards/regions, the density split was determined by the image-level ground-truth fruit count, and the maturity split is defined based on cluster-level maturity categories (Green, Turning-red, and Fully-red) aggregated at the image level. For each subset, the MAE and RMSE were evaluated, accompanied by corresponding statistical visualizations (box plots, radar plots, and line plots) in Fig. 7. In addition, to assess generalization to unseen orchard conditions, we further conducted a cross-orchard validation experiment by training models on Sites a–d and testing them on the held-out Site e.

#### 3.3.2 Region-centered analysis

Test images were first grouped by orchard region (a–e), and the corresponding MAE and RMSE results are summarized in Table S2. Counting performance varied notably across these locations, reflecting underlying differences in canopy structure, illumination, and background complexity. Notably, Regions d and e were the most challenging subsets, where several baseline methods exhibited pronounced performance degradation. For example, BL increased from single-digit errors in Regions a and b (MAE/RMSE: 5.84/7.75 and 6.70/8.82, respectively) to substantially larger errors in Regions d and e (21.58/35.18 and 23.56/45.28, respectively). Similar degradation was also observed for GL and Gramformer in Regions d and e (e.g., GL: 18.18/30.71 and 19.57/42.37; Gramformer: 26.35/43.30 and 18.61/38.31).

In contrast, the proposed method remains stable on these difficult regions, achieving the lowest RMSE in Region d (9.82) and Region e (14.82), while keeping MAE close to the best-performing baseline on both subsets (7.14 in Region d and 11.12 in Region e). This trend is further supported by the radar plot in Fig. 7D–E, where most competing methods shrink noticeably on Regions d and e, whereas our method maintains a more balanced profile with stronger scores on these challenging regions.

#### 3.3.3 Density-centered analysis

The test images were further stratified into three density levels according to the ground-truth fruit count: Low (≤100), Medium (101–150), and High (>150). The corresponding quantitative results are summarized in Table S3. To complement the mean MAE and RMSE metrics, the MAE distributions are visualized via box plots to evaluate error stability (Fig. 7A–C). In each box plot, the box spans the interquartile range (25th–75th percentiles), while the orange dashed and blue dashed lines denote the median and mean MAE, respectively; the proposed method is highlighted in orange.

The proposed method achieved the lowest MAE across all three density levels, with values of 5.71, 7.70, and 11.73 in the low, medium, and high density groups, respectively. It also obtained the lowest RMSE in the medium and high density groups, with values of 9.41 and 14.74, respectively (Table S3). MS2 yielded the lowest RMSE in the Low-density subset (8.54), while our method remains competitive (9.79). In contrast, errors of several baseline methods increased sharply as density grows, e.g., BL rose from 5.85/9.73 (Low) to 20.98/41.07 (High), and GL rose from 6.11/9.62 (Low) to 19.65/39.32 (High). These quantitative trends are consistent with the box plots in Fig. 7A–C. As density increases, our method maintains a more concentrated MAE distribution with a relatively compact IQR and shorter whiskers, suggesting more stable predictions when fruit clusters become dense and occlusions are severe.

#### 3.3.4 Maturity-centered analysis

Finally, maturity-centered counting was evaluated for Green, Turning-red, and Fully-red clusters. For each maturity stage, only the corresponding cluster foreground regions were retained in each test image, while other regions were masked out. The fruit dots within the retained regions were used as ground truth, and MAE and RMSE were computed over the test set.

Quantitative results demonstrate that the proposed method achieved the lowest RMSE across all three maturity subsets (3.15, 21.28, and 36.50 for the Green, Turning-red, and Fully-red stages, respectively) (Table S4). This indicates enhanced counting stability amidst appearance variations across different phenophases. Although the BL model attained the lowest MAE across the three subsets (1.73, 7.02, and 10.44), the proposed method yielded highly competitive MAE values (1.78, 7.31, and 11.26), with only marginal differences during the green and turning-red stages. The advantage of the proposed method was more evident in RMSE, which is more sensitive to large estimation errors. By consistently reducing these large-error cases relative to competing methods, the proposed framework demonstrates stronger robustness to challenging conditions such as green-stage visual ambiguity and partial occlusion. This trend is also supported by Fig. 7F–H, where the proposed method maintains the most favorable RMSE profile across all maturity levels.

#### 3.3.5 Cross-orchard validation

To further evaluate the generalization ability of the proposed method under unseen orchard conditions, we conducted a cross-orchard validation experiment. Specifically, images from Sites a–d were used for training, while all images from Site e were held out for testing. This protocol is more challenging than the random image-level split because the test orchard was completely unseen during model training.

As shown in Table 3, FG-LCNet achieved the best performance among all compared methods, with an MAE of 15.50 and an RMSE of 20.14. Compared with the strongest baseline, DM-Count, the proposed method reduced MAE from 17.89 to 15.50 and RMSE from 24.64 to 20.14. The improvements indicate that the foreground-guided two-stage design remains effective when transferred to an unseen orchard. In particular, the first-stage foreground localization helps reduce background interference, while the second-stage density regression improves counting stability in dense and occluded fruit-cluster regions. Although the errors under this cross-orchard setting are higher than those under the random image-level split, the proposed method still shows better generalization than existing counting baselines.

**Table 3:** Cross-orchard validation results.

Method	MAE *↓*	RMSE *↓*
BL	20.09	43.30
DM-Count	17.89	24.64
GL	30.18	61.71
Gramformer	19.76	38.17
MS2	25.74	59.11
Ours	**15.50**	**20.14**

### 3.4 Ablation studies

Ablation studies were conducted to quantify the contribution of key components in both stages of the proposed framework. Unless otherwise stated, all variants were trained and evaluated under the same protocol as in the main experiments.

#### 3.4.1 Stage-1 detection ablation

The individual and combined effects of the proposed detection refinements (the P2 layer and the GLSA module) were systematically evaluated (Table 4(i)). Interestingly, integrating either component in isolation did not yield consistent performance gains. The P2-only variant slightly reduced *AP*_50_ (0.750 vs. 0.760) but improved HitRate_green_ (63.09% vs. 62.02%), whereas the GLSA-only variant yielded a comparable *AP*_50_ (0.758) but a lower HitRate_green_ (60.96%). In contrast, jointly incorporating P2 and GLSA resulted in the best performance, increasing *AP*_50_ from 0.760 to 0.769 and improving HitRate_green_ from 62.02% to 68.07%. These results suggest that P2 and GLSA are complementary rather than independently additive. P2 preserves high-resolution details for small or distant litchi clusters but may also introduce confusing foliage and background textures, whereas GLSA suppresses background interference through global–local contextual filtering but cannot fully compensate for missing fine-grained details. Their combination therefore balances sensitivity to small litchi clusters and background suppression, explaining why the joint variant performs best while either module alone yields limited or inconsistent gains.

**Table 4:** Stage-1 ablation and threshold sensitivity results.

(i) Module ablation		
Variant	*AP*_50_*↑*	HitRate_green_*↑*
Baseline (w/o P2 & GLSA)	0.760	62.02%
+ P2 only	0.750	63.09%
+ GLSA only	0.758	60.96%
+ P2 & GLSA (Ours)	**0.769**	**68.07%**

(ii) Sensitivity to confidence threshold		
Confidence threshold *τ*	MAE *↓*	RMSE *↓*
0.05	9.52	14.44
0.10	8.36	12.95
0.25 (Ours)	**7.44**	**11.01**
0.40	11.63	19.76
0.50	17.08	30.28

#### 3.4.2 Sensitivity to the Stage-1 detection confidence threshold

As shown in Table 4(ii), both overly low and overly high confidence thresholds degraded counting performance. Lower thresholds such as *τ* = 0.05 and *τ* = 0.10 retained more low-confidence proposals, which may introduce noisy foreground regions into Stage 2. In contrast, higher thresholds such as *τ* = 0.40 and *τ* = 0.50 substantially increased the counting errors, likely because small or visually ambiguous fruit clusters were filtered out before density regression. The default setting *τ* = 0.25 achieved the best MAE and RMSE among the evaluated thresholds and was therefore used in all experiments.

#### 3.4.3 Stage-2 ablations on density regression

Component ablations of the Stage-2 counting module are presented in Table 5(i). Removing the hybrid attention mechanism led to noticeable performance degradation (MAE 8.75, RMSE 12.34), suggesting that global–local feature modeling is critical for resolving dense fruit clusters and severe partial occlusions. A similar trend was observed when the consistency loss was removed, which increased the errors to MAE 8.30 and RMSE 11.65. This result suggests that consistency regularization contributes to more stable density estimation under complex background conditions. The full model achieved the best overall performance, with an MAE of 7.44 and an RMSE of 11.01.

**Table 5:** Stage-2 ablations on counting.

Variant	MAE *↓*	RMSE *↓*
**(i) Main component ablation**		
w/o hybrid attention	8.75	12.34
w/o consistency loss	8.30	11.65
Ours	**7.44**	**11.01**

**(ii) Branch ablation of hybrid attention**		
w/o self-attention branch	8.85	16.77
w/o conv-attention branch	9.05	16.85
Ours	**7.44**	**11.01**

**(iii) Number of hybrid-attention blocks**		
× 2	7.70	**10.99**
× 3 (ours)	**7.44**	11.01
× 4	8.49	12.56
× 5	9.13	13.26

#### 3.4.4 Ablation of the hybrid attention module

We further analyzed the Hybrid Attention Module in terms of branch design and block number. For branch-level ablation (Table 5(ii)), removing the self-attention branch increased the errors to 8.85 MAE and 16.77 RMSE, while removing the convolutional-attention branch resulted in 9.05 MAE and 16.85 RMSE. These results indicate that the two branches are complementary: convolutional attention enhances local texture and spatial–channel responses, whereas self-attention captures broader contextual dependencies in dense clusters.

For block-number ablation (Table 5(iii)), two blocks achieved a slightly lower RMSE but a higher MAE, whereas three blocks provided the best overall trade-off with the lowest MAE and comparable RMSE. Increasing the number of blocks to 4–5 degraded performance, likely due to over-parameterization and optimization difficulty. Therefore, three Hybrid Attention Blocks were used as the default setting.

### 3.5 Runtime analysis and practical deployment

Agricultural applications require not only accurate counting but also sufficiently fast inference for in-field use and responsive human–computer interaction. Although FG-LCNet is not explicitly optimized for lightweight deployment through techniques such as pruning, quantization, or architecture compression, and therefore incurs a relatively higher inference cost than some simpler baselines, it still satisfies practical runtime requirements of real-world use on a single commodity GPU. Considering its stronger and more robust counting performance, this additional computational overhead remains acceptable for real-world orchard applications.

#### 3.5.1 Evaluation setup

Inference efficiency was benchmarked on an NVIDIA RTX 3090 GPU with a batch size of 1. All models took a single 512 × 512 RGB image as input. Efficiency was quantified in terms of parameter size and per-image inference latency (ms), with frames per second (FPS) calculated as 1000/latency. Unless otherwise stated, the reported latency was averaged over multiple repeated runs with warm-up under the same implementation settings.

#### 3.5.2 Runtime and model complexity

The runtime and model-size statistics of the compared Stage-1 detectors and end-to-end counting models confirm the practical viability of the proposed approach (Table 6). For Stage-1 cluster detection, the proposed detector contains 3.84M parameters and operates at 5.29 ms/image (188.97 FPS). While marginally slower than the YOLO baselines, it remains substantially faster than RT-DETR (16.07 ms/image, 62.24 FPS). This indicates that the improved detection performance (Table 2(i)) is achieved with a moderate and still practical computational cost.

**Table 6:** Runtime and model complexity on RTX 3090.

Method	Params (M) *↓*	Latency (ms/img) *↓*	FPS *↑*
**Stage-1: cluster detection**			
YOLOv8	2.69	2.80	357.75
YOLOv10	2.71	3.53	283.28
YOLOv11	2.59	3.43	291.38
RT-DETR	8.10	16.07	62.24
Ours	3.84	5.29	188.97

**End-to-end counting**			
BL	21.5	9.77	102.39
DM-Count	21.5	9.77	102.39
GL	21.5	9.77	102.39
Gramformer	29.01	12.89	77.57
MS2	45.88	15.36	65.11
Ours	25.89	21.78	45.91

For the complete counting task, the proposed two-stage pipeline contains 25.89M parameters and requires 21.78 ms per image, corresponding to 45.91 FPS on an RTX 3090 GPU. The measured latency covers the full inference process, including Stage-1 detection, bounding-box merging, foreground masking, Stage-2 density regression, and final count aggregation. Its latency is higher than that of several end-to-end counting baselines, which is expected because the framework does not specifically target lightweight acceleration and performs counting through a detection-plus-density estimation pipeline. Notably, BL, DM-Count, and GL report identical model size and inference speed because they share the same backbone architecture and differ mainly in training objectives. Nevertheless, the overall response speed remains sufficient for practical interactive use. Considering its more robust counting performance, this trade-off between efficiency and accuracy is reasonable for deployment in real orchard scenarios.

#### 3.5.3 Mini-program deployment

**Fig. 8:**
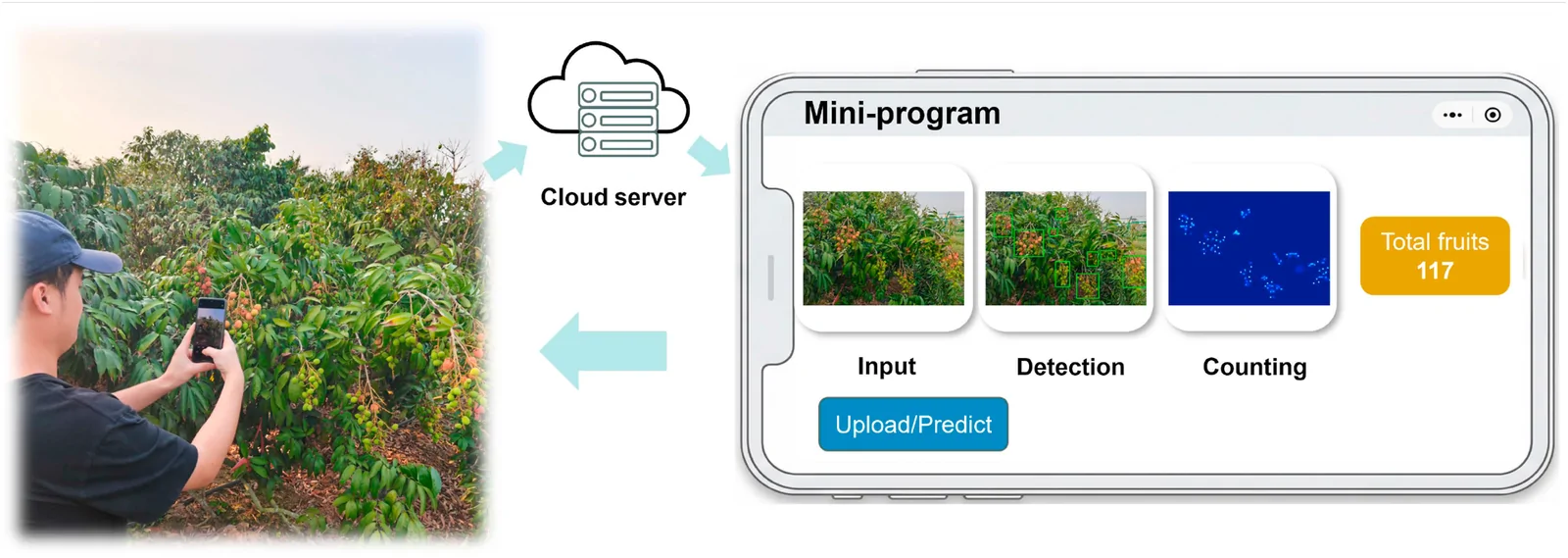
Deployment demonstration of the proposed framework in a mini-program interface.

To facilitate real-world adoption, the proposed framework has been integrated into a practical inference service and has entered pilot testing through a mini-program interface. Users can upload orchard images and obtain fruit-count predictions together with visual feedback, including detected fruit clusters and density-map overlays. Although the method is not aggressively optimized for minimal latency, the current inference speed is already sufficient to support smooth interactive use in practice. The deployment workflow and example interface are shown in Fig. 8.

## 4 Conclusion

In this study, we presented FG-LCNet, a foreground-guided two-stage framework for litchi counting in real orchard environments. By decoupling fruit-cluster localization from within-cluster counting, the method is better suited to dense distribution, heavy occlusion, and stage-dependent appearance changes in whole-tree litchi images than conventional single-stage strategies. The improved detector enhances sensitivity to small and ambiguous clusters, while the hybrid-attention density-regression network and consistency-based supervision improve robustness to background interference, illumination variation, and maturity-related appearance shifts. In addition, we constructed a large-scale litchi counting dataset with fruit-level dot annotations and cluster-level bounding boxes, covering multiple orchards, diverse cultivars, and three ripening stages, which provides a valuable benchmark for robust litchi counting and related plant phenotyping research.

Experimental results show that FG-LCNet achieves strong overall counting accuracy, with its most evident advantages observed in high-density fruit-cluster scenarios and cross-orchard validation. The method also shows competitive performance across orchard-region and maturity-stage subsets, while maintaining practical inference efficiency for interactive deployment. These findings suggest that introducing explicit foreground priors before density estimation is an effective strategy for improving counting reliability in complex orchard scenes. Beyond litchi, the proposed framework may also be useful for other clustered fruit-counting tasks under severe occlusion and appearance variation.

Several limitations remain. First, although the dataset is diverse, green-stage samples are still relatively underrepresented, which may constrain performance in the earliest and most challenging phenophase. Second, as a two-stage pipeline, the framework may still suffer from error propagation from detection to counting. Third, the current study focuses on visible fruit counting from single-view whole-tree images rather than complete 3D fruit-load estimation. Finally, the current model has not been specifically optimized for lightweight edge deployment on mobile or embedded devices. Future work will focus on improving green-stage robustness, reducing cross-stage and cross-orchard domain gaps, exploring multi-view counting for full-tree yield estimation, developing lighter implementations for field use, and evaluating the framework under stricter generalization settings such as orchard-level or season-level splits.

## Author contributions

Y. Zhuang designed and conducted the experiments, reorganized and reclassified the dataset, performed data visualization, and drafted and revised the manuscript. M. Yang conceived the idea, acquired funding and provided project administration, collected, organized, and labeled the dataset, performed data visualization, and participated in drafting and revising the manuscript. Z. Ke conducted the experiments, reorganized and reclassified the dataset, and participated in drafting. R. Chen conceived the idea, performed data visualization, provided project administration and offered technical support. Y. Gao provided project administration and offered technical support. X. Wang and F. Hu provided project administration and funding support.

## Data availability

The implementation code is publicly available at https://github.com/johnhamtom/FG-LCNet. Upon acceptance, a representative subset of approximately 100 annotated litchi images will be released to support reproducibility and preliminary benchmarking. The full dataset is being further organized for future release. Before full release, the complete dataset can be obtained from the corresponding author, M. Yang, upon reasonable request.

## Funding

This work was supported by 10.13039/501100004761Hainan Provincial Natural Science Foundation of China (325QN478), 10.13039/501100008111Hainan Provincial Science and Technology Talent Innovation Program (KJRC2023C17), the China Litchi and Longan Industry Technology Research System (CARS-32-21), 10.13039/501100015335Hainan Modern Agricultural Industry Technology System (HNARS-08-G02).

## Declaration of interest statement

The authors declare that they have no known competing financial interests or personal relationships that could have appeared to influence the work reported in this paper.
