# Supplementary material for: FG-LCNet: A two-stage foreground-guided network for whole-tree litchi counting
Source: Plant Phenomics. 2026 Sep 7;8(4):100268. doi: 10.1016/j.plaphe.2026.100268 (PMC13584049; doi:10.1016/j.plaphe.2026.100268)
Supplement: Supplementary file 1 [file mmc1.pdf]

Supplementary Materials

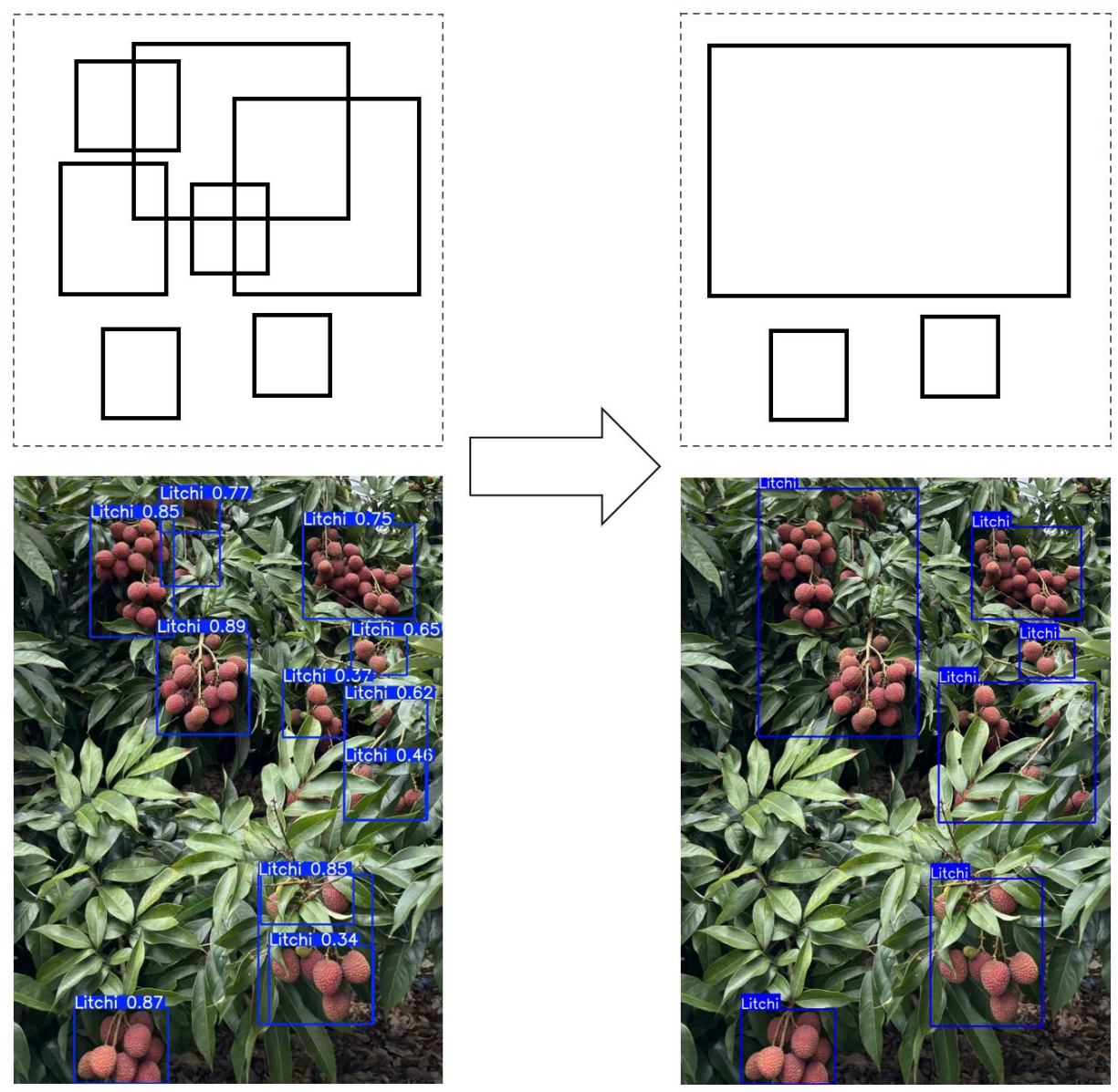

Fig. S1. Bounding-box merging strategy.

Table S1. Dataset split for training, validation, and testing.

**Subset**	**Image Count (n)**	**Dot Annotations (n)**	**Bounding Boxes (n)**
Training	675	73,493	12,812
Validation	225	22,768	4,158
Testing	226	24,051	4,165

Table S2. Stratified counting results by orchard region.

Method	Region a		Region b		Region c		Region d		Region e	
	MAE ↓	RMSE ↓	MAE ↓	RMSE ↓	MAE ↓	RMSE ↓	MAE ↓	RMSE ↓	MAE ↓	RMSE ↓
BL	**5.84**	**7.75**	**6.70**	**8.82**	4.62	7.41	21.58	35.18	23.56	45.28
DM-Count	9.50	12.43	9.43	14.38	5.99	9.99	**6.98**	9.89	**11.09**	15.86
GL	6.30	8.20	7.93	11.88	7.93	11.8	18.18	30.71	19.57	42.37
Gramformer	6.53	8.60	6.80	10.02	**3.71**	**6.32**	26.35	43.30	18.61	38.31
MS2	8.63	11.00	9.11	11.40	5.65	10.22	16.80	25.04	20.96	39.78
Ours	7.01	9.23	7.91	13.44	5.09	7.87	7.14	**9.82**	11.12	**14.82**

Table S3. Stratified counting results by fruit density.

Method	Low (≤ 100)		Medium (101–150)		High (> 150)	
	MAE ↓	RMSE ↓	MAE ↓	RMSE ↓	MAE ↓	RMSE ↓
BL	5.85	9.73	9.56	15.77	20.98	41.07
DM-Count	6.32	9.81	11.40	14.44	12.80	17.27
GL	6.11	9.62	8.56	13.36	19.65	39.32
Gramformer	5.97	10.96	9.36	16.61	19.79	38.62
MS2	6.63	**8.54**	11.00	14.87	21.39	37.13
Ours	**5.71**	9.79	**7.70**	**9.41**	**11.73**	**14.74**

Table S4. Stratified counting results by maturity stage. Lower is better.

Method	Green		Turning-red		Fully-red	
	MAE ↓	RMSE ↓	MAE ↓	RMSE ↓	MAE ↓	RMSE ↓
BL	**1.73**	3.52	**7.02**	22.53	**10.44**	37.21
DM-Count	2.24	3.83	8.16	21.72	12.78	37.19
GL	1.75	3.81	7.79	22.68	10.91	37.47
Gramformer	3.27	5.60	9.04	23.86	13.75	39.01
MS2	3.86	5.73	11.24	24.55	16.61	40.02
Ours	1.78	**3.15**	7.31	**21.28**	11.26	**36.50**
